# The additional benefit of computed tomography in cancer patients: impacts of sarcopenia and cachexia on quality of life during chemotherapy

**DOI:** 10.1590/0100-3984.2024.0012

**Published:** 2024-08-02

**Authors:** Galtieri Otávio Cunha de Medeiros, Ilanna Marques Gomes da Rocha, Aline Marcadenti, Ricardo Andrade Bezerra, Erica Roberta Barbalho, Carlos Alves de Sousa Júnior, Ana Paula Trussardi Fayh

**Affiliations:** 1 Universidade Federal do Rio Grande do Norte (UFRN), Natal, RN, Brazil; 2 Instituto de Pesquisa do Hospital do Coração (IP-HCor), São Paulo, SP, Brazil; 3 Universidade Federal de Ciências da Saúde de Porto Alegre (UFCSPA), Porto Alegre, RS, Brazil

**Keywords:** Gastrointestinal neoplasms, Cachexia, Sarcopenia, Drug therapy, Antineoplastic agents/adverse effects, Quality of life, Neoplasias gastrointestinais, Caquexia, Sarcopenia, Tratamento farmacológico, Antineoplásicos/efeitos adversos, Qualidade de vida

## Abstract

**Objective:**

This study evaluates the effects of sarcopenia and cachexia on the quality of life (QoL) of patients with gastrointestinal cancer during their initial cycle of chemotherapy, emphasizing the significance of computed tomography (CT) in assessing muscle mass.

**Materials and Methods:**

In this prospective study, we evaluated 60 adult patients with gastrointestinal cancer who started chemotherapy between January and December of 2017. Sarcopenia was diagnosed on the basis of CT findings, and QoL was assessed with the European Organization for Research and Treatment of Cancer Quality of Life Questionnaire Core 30.

**Results:**

The mean age was 60.9 years, and 33 (55.0%) of the patients were men. Of the 60 patients, 33 (55.0%) had cachexia and 14 (23.3%) had sarcopenia. Chemotherapy significantly reduced QoL, particularly in the physical, role functioning, and social domains, with no differences between the cachexia and sarcopenia groups.

**Conclusion:**

Among patients with gastrointestinal cancer submitted to chemotherapy, the chemotherapy-induced decline in QoL does not seem to differ significantly between those with cachexia or sarcopenia, as classified by CT-measured muscle mass, and those without. However, CT-based muscle mass evaluation remains crucial for guiding customized intervention strategies. Integrating this evaluation in radiological reports can provide valuable insights for planning specific care, thus improving patient QoL during treatment.

## Abstract (pt)

**Objetivo:**

Este estudo avalia os efeitos da sarcopenia e da caquexia na qualidade de vida de pacientes com câncer gastrointestinal durante o ciclo inicial de quimioterapia, enfatizando a importância da tomografia computadorizada (TC) na avaliação da massa muscular.

**Materiais e Métodos:**

Estudo prospectivo com 60 pacientes adultos com câncer gastrointestinal que iniciaram quimioterapia de janeiro a dezembro de 2017. A TC foi utilizada para o diagnóstico de sarcopenia e o Quality of Life Questionnaire Core 30 da European Organization for Research and Treatment of Cancer foi utilizado para avaliar a qualidade de vida.

**Resultados:**

A média de idade dos pacientes foi 60,9 anos e 33 (55%) eram homens. Entre os pacientes, 33 (55%) eram caquéticos e 14 (24%) eram sarcopênicos. A quimioterapia reduziu significativamente a qualidade de vida, especialmente nos domínios físico, de desempenho de papéis e social, sem diferenças entre os grupos caquéticos e sarcopênicos.

**Conclusão:**

A diminuição da qualidade de vida não difere significativamente entre pacientes caquéticos/sarcopênicos e não caquéticos/não sarcopênicos com câncer gastrointestinal submetidos a quimioterapia, conforme classificado pela massa muscular medida por TC. No entanto, a avaliação da massa muscular por TC continua crucial para orientar estratégias de intervenção personalizadas. A integração dessa avaliação nos laudos radiológicos pode fornecer informações valiosas para o planejamento de cuidados específicos, melhorando a qualidade de vida dos pacientes durante o tratamento.

## INTRODUCTION

Sarcopenia, defined as the progressive and generalized loss of skeletal muscle mass and strength, is a condition often observed in cancer patients, adversely affecting quality of life (QoL) and prognosis^(1)^. Cachexia, on the other hand, is a complex metabolic syndrome, characterized by severe weight loss, muscle atrophy, fatigue, and weakness, that is not fully reversible by conventional nutrition^(2)^. Although distinct in their definitions and diagnostic criteria, both conditions are critical comorbidities in cancer, influencing treatment efficacy, survival, and patient QoL. This distinction and the clinical relevance of each condition justify the need for accurate assessment, hence the importance of computed tomography (CT) for the objective measurement of muscle mass.

In recent years, considerable progress in diagnosis and treatment has contributed to a significant improvement in prognosis and increase in survival among cancer patients. Consequently, patient QoL is becoming more and more important, and its evaluation is of increasing interest^(3)^. Functional disorders that arise during or after treatments such as chemotherapy can lead to a range of side effects, including nausea, vomiting, diarrhea, constipation, mucositis, and fluctuations in weight or hormone levels^(4)^. Despite these developments, no studies have examined the short-term impact of treatment on the QoL of cancer patients. Dahiya et al.^(5)^ evaluated the QoL of 67 newly diagnosed women with advanced cervical cancer after six months of treatment and observed a significant improvement following chemoradiotherapy. However, the impact of treatment on QoL during chemotherapy alone was not evaluated.

The treatment for gastrointestinal cancer can often lead to significant weight loss and malnutrition^(6)^. Malnutrition among cancer patients is known to correlate with diminished overall well-being and performance, increased fatigue, and lower tolerance to treatments^(7)^, whereas chemotherapy and radiotherapy could both have adverse effects on patient nutritional status and functional health^(8)^. Sarcopenia and cachexia are closely associated with a decline in functional performance and diminished participation in everyday activities^(9)^. However, it has yet to be well established how nutritional status is determined, especially in the setting of sarcopenia and cachexia, or how it can affect the QoL of cancer patients during treatment.

The use of anticancer drugs plays a significant role in the occurrence of adverse events during chemotherapy, a topic that was extensively reported on by Daly et al.^(10)^. Unlike the side effects experienced in a clinical setting, the adverse effects faced by outpatients undergoing cancer chemotherapy can directly impact their home and work life, potentially leading to alterations in their QoL^(11)^.

Contributing to all of this scenario, CT is seen as an opportunist tool for evaluating muscle mass, because these examinations are initially performed when there is a clinical indication for cancer staging. Consequently, these images are utilized for the timely assessment of body composition. From that viewpoint, the aim of this study is to explore the effects of sarcopenia and cachexia on the QoL of patients with gastrointestinal cancer during their first cycle of chemotherapy, highlighting the additional advantages offered by the CT assessment of muscle mass.

## MATERIALS AND METHODS

### Study design and participant selection

This was a prospective study, conducted from January to December 2017, focusing on adult and elderly patients with gastrointestinal cancer at a single center. The criteria for inclusion were starting chemotherapy (either neoadjuvant or adjuvant) and having a biopsy-confirmed diagnosis of gastrointestinal cancer. Patients with significant cognitive impairments or serious psychiatric conditions were excluded. The characteristics of the sample, including chemotherapy toxicities, are detailed in a previous study^(12)^, which describes the association between cachexia and chemotherapy toxicities in gastrointestinal cancer patients. The present study was conducted in accordance with the Declaration of Helsinki guidelines and was approved by the Human Research Ethics Committee (Reference no. 64765517.0.0000.5292).

The calculation for the required number of study participants was based on previous research that analyzed the link between sarcopenia and the maximum tolerable dose toxicity during a single chemotherapy session for 72 patients with operable esophageal cancer. This analysis aimed to achieve a statistical power of 80% at a significance threshold of 0.05, using G*Power software, version 3.1.9.2 (Institute for Experimental Psychology, Dusseldorf, Germany). We thus calculated that the minimum sample size would be 36 patients.

### Procedures

Patients who met the inclusion criteria were monitored during their initial chemotherapy cycle, irrespective of the cycle length, which was determined by the physician for the specific type of cancer. Demographic, disease, clinical, and pathology data were gathered from the electronic medical records of the hospital.

On the first day of each chemotherapy cycle, patients underwent nutritional and functional evaluations. The body mass index (BMI) was calculated from height and weight measurements. The patients were thus categorized as underweight, normal weight, overweight, or obese in accordance with the World Health Organization guidelines^(13)^. Functional capacity was evaluated at the start of each chemotherapy cycle using handgrip strength and the Eastern Cooperative Oncology Group-Performance Status scale, on which a score of ≥ 2 was considered indicative of low functional capacity^(14)^.

### QoL assessment

The QoL of each study participant was evaluated before and after their initial cycle of chemotherapy with the aid of the questionnaire formulated by the European Organization for Research and Treatment of Cancer Quality of Life Questionnaire Core 30 (EORTC QLQ-C30). The EORTC QLQ-C30 is a recognized tool for assessing cancer-specific QoL, created in 1993 for general cancer patient populations^(15)^. It is a 30-item questionnaire specific to cancer, employed to gauge patient symptoms, functionality, and QoL, including five functional scales (physical, emotional, cognitive, social, and role), three symptom scales (fatigue, pain, nausea or vomiting), and six individual items evaluating symptoms and their functional effect on the illness, as well as providing a global health/QoL score. Higher scores on the functional scales signify improved functioning, whereas higher scores on the symptom scales and individual items indicate more pronounced symptoms or greater functional detriment. The EORTC QLQ-C30 has been translated to several languages, including Portuguese^(16)^, validated for use in the corresponding cultures, and utilized in a multitude of studies worldwide.

### Sarcopenia and cachexia assessment

**Figure 1:**
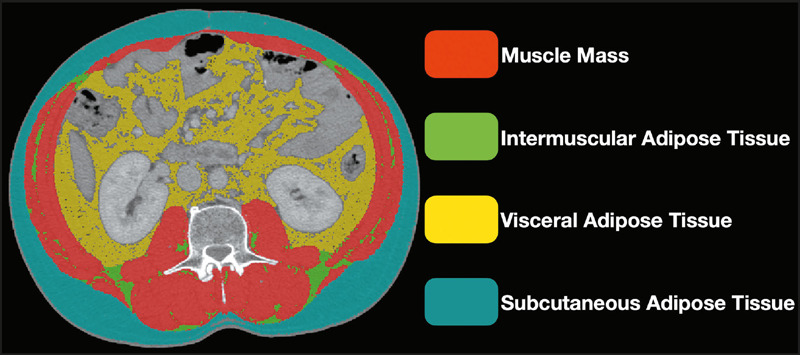
Segmentation of the rectus abdominis, transversus abdominis, internal oblique, external oblique, psoas, quadratus lumborum, erector spinae, and latissimus dorsi muscles at the level of the third lumbar vertebra.

To evaluate muscle mass, muscle area (cm^2^) was measured on CT scans performed for diagnostic purposes (Figure 1), typically approximately 30 days prior to the start of chemotherapy. The third lumbar vertebra served as the standard reference point, and was semi-automatically segmented, with muscle tissue identified in the range of -29 to +150 HU^(17)^, with Slice-O-matic Software, version 5.0 (Tomovision, Quebec, Canada). Established thresholds— skeletal muscle index (SMI) of less than 43 cm^2^/m^2^ in men with a BMI < 25 kg/m^2^, below 53 cm^2^/m^2^ in men with a BMI ≥ 25 kg/m^2^, and under 41 cm^2^/m^2^ in women—were applied to identify low muscle mass^(18)^.

Handgrip strength was gauged with a hydraulic hand dynamometer (Jamar, Mississauga, Canada). Each hand was tested alternately for three attempts, each lasting a minimum of three seconds, and the highest value recorded was taken as the maximum muscle strength. Dynapenia was classified when handgrip strength < 30 kg and < 20 kg for men and women, respectively, and patients with dynapenia and low muscularity were considered sarcopenic^(19)^. Cachexia was classified as described by Fearon et al.^(20)^, evaluating involuntary weight loss, BMI, and muscle mass.

### Statistical analyses

The data were analyzed with the IBM SPSS Statistics software package for Windows, version 22.0 (IBM Corp., Armonk, NY, USA). The normality of the data was checked with the Kolmogorov-Smirnov test, and nonparametric data were converted using the log function for parametric analysis. Descriptive statistics are presented as means ± standard deviations (SDs), as medians (ranges), or as absolute and relative frequencies. Comparisons of QoL between patients with and without sarcopenia and between those with and without cachexia were made by using independent t-tests for unpaired data. The difference between the means of the global health score before and after treatment (delta) was assessed. Analysis of covariance was employed to assess the difference in delta values of the global health score based on the presence or absence of sarcopenia and cachexia, adjusting for the type of treatment and stage of the disease. Statistical significance was set at *p* < 0.05.

## RESULTS

**Figure 2:**
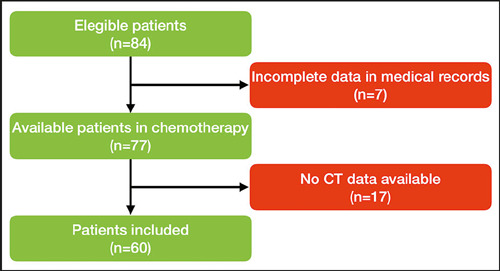
Flow chart of the patient selection process.

Although 77 individuals met the inclusion criteria and commenced chemotherapy during the study period, only 60 had accessible CT scans. Figure 2 summarizes the patient inclusion process.

Table 1 describes the baseline demographic characteristics of the patients, chemotherapy regimens, and nutritional characteristics. The mean age of the patients was 60.9 ± 14.0 years, and 33 (55.0%) were men. Colorectal cancer was the most common type of cancer encountered (36 patients; 60.0%), followed by stomach cancer (14 patients; 23.3%). In terms of nutritional status, 35 patients (58.3%) were classified as malnourished, based on the Patient-Generated Subjective Global Assessment, with categories B and C indicating a risk of malnutrition. Despite many patients having a normal weight (26 patients; 43.3%), 33 (55.0%) satisfied the criteria for cachexia and 14 (24.0%) satisfied the criteria for sarcopenia.

**Table 1:** Clinical and nutritional characteristics of the patients at baseline.

Characteristic	(N = 60)
Age (years), mean ± SD	60.9 ± 14.0
≤ 60 years, n (%)	27 (45.0)
> 60 years, n (%)	33 (55.0)
Sex, n (%)	
Female	27 (45.0)
Male	33 (55.0)
Ethnicity, n (%)	
White	18 (30.0)
Non-White	42 (70.0)
Tumor site, n (%)	
Esophagus	6 (10.0)
Stomach	14 (23.3)
Colon/rectum	36 (60.0)
Other gastrointestinal	4 (6.7)
Clinical TNM stage, n (%)	
II	8 (13.3)
III	24 (40.0)
IV	28 (46.7)
Treatment modalities, n (%)	
Chemotherapy	12 (20.0)
Chemotherapy + radiotherapy	21 (35.0)
Chemotherapy + surgery	20 (33.3)
Chemotherapy + radiotherapy + surgery	7 (11.7)
Chemotherapy protocol, n (%)	
5FU + leucovorin	36 (46.7)
FOLFOX	12 (15.6)
Paclitaxel + carboplatin	11 (14.3)
Other	4 (5.2)
Height (m), mean ± SD	1.60 ± 0.09
Weight (kg), mean ± SD	61.5 ± 14.9
BMI (kg/m^2^), mean ± SD	24.5 ± 5.7
Underweight, n (%)	6 (10.0)
Normal weight, n (%)	26 (43.3)
Overweight, n (%)	19 (31.7)
Obese, n (%)	9 (15.0)
SMI (cm^2^/m^2^), mean ± SD	
Men	53.5 ± 10.1
Women	46.4 ± 8.4
Muscle attenuation (HU), mean ± SD	37.8 ± 9.1
Low muscle attenuation, n (%)	30 (50.0)
Sarcopenia, n (%)	14 (23.3)
PG-SGA, n (%)	
Nourished	25 (41.7)
Malnourished	35 (58.3)
ECOG performance status, n (%)	
0–1	47 (78.3)
2	13 (21.7)
EORTC QLQ-C30 scores, mean ± SD	
Global health	73.3 ± 18.4
Physical functioning	80.0 ± 14.9
Role functioning	72.0 ± 22.3
Emotional functioning	85.7 ± 16.5
Cognitive functioning	88.3 ± 14.1
Social functioning	77.5 ± 20.3

TNM, tumor-node-metastasis; PG-SGA, patient-generated subjective global assessment; ECOG, Eastern Cooperative Oncology Group.

Pre-chemotherapy QoL scores indicated moderate levels across the domains, with a mean global health score of 73.3. Table 1 details the mean EORTC QLQ-C30 scores for physical functioning (80.0), role functioning (72.0), emotional functioning (85.7), cognitive functioning (88.3), and social functioning (77.5). Of the 60 patients evaluated, 45 (75%) experienced some level of self-reported toxicity during their chemotherapy.

In the assessment of muscle mass via CT, our study focused on the cross-sectional area at the third lumbar vertebra level. This approach has been validated for its precision in measuring skeletal muscle mass, with the area adjusted for patient height to calculate the SMI. Based on Table 1, the study population presented a wide range of clinical and nutritional characteristics, which significantly influenced the interpretation of muscle area measurements.

The mean SMI values were 53.5 cm^2^/m^2^ for men and 46.4 cm^2^/m^2^ for women. This condition was notably more common among the patients over 60 years of age (55.0% of our study population), who also exhibited lower muscle attenuation values (mean, 37.8 HU). Notably, 50% of our patients had low muscle attenuation, further substantiating the critical association between sarcopenia, cachexia, and adverse clinical outcomes in gastrointestinal cancer.

**Figure 3:**
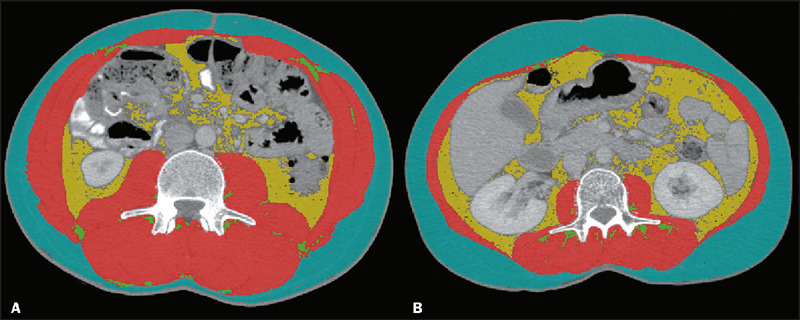
Comparison between patients with and without sarcopenia. **A:** Male patient without sarcopenia (BMI, 23.46 kg/m^2^; muscle area, 201.6 cm^2^). **B:** Male patient with sarcopenia (BMI, 21.91 kg/m^2^; muscle area, 84.2 cm^2^).

Sarcopenia was identified in 14 (23.3%) of the patients (Figure 3), aligning with the recognized impact of gastrointestinal cancer on muscle degradation. These findings are critical, as they highlight the urgent need for integrating muscle mass evaluation into the standard clinical assessment of cancer patients.

Patients with cachexia reported moderate levels of QoL before and after chemotherapy, showing a significant difference between the beginning and the end of the chemotherapy cycle (*p* = 0.001). However, there were no discernible differences in QoL between those with and without cachexia (Table 2). Despite a general decline in all domains by the end of the cycle compared to the beginning, were found statistically significant changes in physical functioning (*p* = 0.039), role functioning (*p* = 0.038), and social functioning (*p* = 0.006). Likewise, no notable differences in QoL were found between patients with and without sarcopenia. While there were declines in most EORTC QLQ-C30 domains (Table 3), only the global health score and the social functioning score exhibited statistically significant differences between the beginning and the end of the chemotherapy cycle (*p* = 0.036 and *p* = 0.05, respectively). Table 4 provides a detailed overview of the variation in QoL and symptom parameters across different levels of decline. The most notable declines were observed in the role functioning and social functioning domains.

**Table 2:** Association between cachexia and QoL during the first chemotherapy cycle (N = 60).

EORTC QLQ-C30 scores	With cachexia (n = 31)				Without cachexia (n = 27)			
	Pre-chemo*	Post-chemo*	95% CI	P^†^	Pre-chemo*	Post-chemo*	95% CI	P^†^
Global health	66.67	66.67		**0.001**	66.67	66.67		
Log global health	1.85 ± 0.09	1.76 ± 0.15	0.046 to 0.133		1.85 ± 0.09	1.79 ± 0.12	0.019 to 0.089	**0.040**
Physical functioning	80.00	73.33		0.039	86.67	73.33		
Log physical functioning	1.87 ± 0.12	1.80 ± 0.22	0.003 to 0.135		1.89 ± 0.11	1.85 ± 0.14	-0.028 to 0.109	0.235
Role functioning	66.67	50.00		**0.038**	83.33	66.70		
Log role functioning	1.77 ± 0.21	1.67 ± 0.26	0.005 to 0.187		1.85 ± 0.17	1.80 ± 0.19	-0.046 to 0.145	0.296
Emotional functioning	83.33	75.00		0.101	83.33	83.33		
Log emotional functioning	1.90 ± 0.11	1.83 ± 0.24	-0.013 to 0.148		1.88 ± 0.09	1.88 ± 0.14	-0.068 to 0.061	0.911
Cognitive functioning	83.33	83.33		0.388	100. 00	83.33		
Log cognitive functioning	1.90 ± 0.13	1.83 ± 0.24	-0.035 to 0.088		1.92 ± 0.14	1.89 ± 0.16	-0.025 to 0.057	0.435
Social functioning	66.67	50.00		**0.006**	83.33	66.67		
Log social functioning	1.83 ± 0.15	1.69 ± 0.24	0.043 to 0.231		1.90 ± 0.13	1.76 ± 0.21	0.068 to 0.211	**0.001**

* Results expressed as means of the scores or as means and SDs of the log values. ^†^ t-test for paired samples (applied to log variables). Pre-chemo, before the first cycle of chemotherapy; Post-chemo, after the first cycle of chemotherapy; CI, confidence interval.

**Table 3:** Association between sarcopenia and QoL during the first chemotherapy cycle (N = 60).

EORTC QLQ-C30 scores	With cachexia (n = 31)				Without cachexia (n = 27)			
	Pre-chemo*	Post-chemo*	95% CI	P^†^	Pre-chemo*	Post-chemo*	95% CI	P^†^
Global health	83.3	66.67			66.67	66.67		
Log global health	1.86 ± 0.13	1.81 ± 1.13	0.004 to 0.106	**0.036**	1.85 ± 0.08	1.77 ± 0.14	0.045 to 0.113	**0.001**
Physical functioning	86.67	73.33			83.33	73.33		
Log physical functioning	1.90 ± 0.11	1.84 ± 0.16	-0.034 to 0.153	0.197	1.88 ± 0.12	1.82 ± 0.20	0.000 to 0.110	**0.050**
Role functioning	66.67	50.00			66.67	66.67		
Log role functioning	1.75 ± 0.24	1.67 ± 0.26	-0.073 to 0.223	0.296	1.82 ± 0.18	1.75 ± 0.23	0.001 to 0.148	**0.047**
Emotional functioning	83.33	83.33			83.33	75.00		
Log emotional functioning	1.85 ± 0.13	1.83 ± 0.23	-0.081 to 0.130	0.630	1.90 ± 0.09	1.86 ± 0.19	-0.023 to 0.101	0.218
Cognitive functioning	75.00	66.66			100	83.33		
Log cognitive functioning	1.86 ± 0.13	1.83 ± 0.18	-0.046 to 0.121	0.355	1.92 ± 0.13	1.90 ± 0.15	-0.026 to 0.060	0.436
Social functioning	75.00	50.00			66.67	50.00		
Log social functioning	1.91 ± 0.10	1.74 ± 0.26	0.000 to 0.340	**0.050**	1.85 ± 0.15	1.72 ± 0.22	0.030 to 0.067	**0.001**

* Results expressed as means of the scores or as means and SDs of the log values. ^†^ t-test for paired samples (applied to log variables). Pre-chemo, before the first cycle of chemotherapy; Post-chemo, after the first cycle of chemotherapy; CI, confidence interval.

**Table 4:** Variation in QoL and symptoms parameters (N = 60).

Parameter	Post-chemotherapy decline in QoL		
	< 10%	10–20%	> 20%
	n (%)	n (%)	n (%)
Global health	19 (31.7)	16 (26.7)	25 (41.6)
Physical functioning	18 (30.0)	18 (30.0)	24 (40.0)
Role functioning	13 (21.7)	9 (15.2)	38 (63.1)
Emotional functioning	19 (31.7)	12 (20.0)	29 (48.3)
Cognitive functioning	34 (56.7)	4 (6.7)	22 (36.6)
Social functioning	12 (20.0)	8 (13.3)	40 (66.7)

Self-reported QoL following chemotherapy (Table 5) showed that global health decreased moderately (by 10 20%) in 16 patients and markedly (by ≥ 20%) in another 25 patients. The most common major (> 20%) alterations in QoL were in the role functioning and social functioning domains. After adjusting for the treatment type and disease stage, we found no statistical difference between the mean global health score delta for patients with cachexia and that observed for those without the disease (-15.39 ± 3.52 vs. -12.68 ± 3.90; *p* = 0.61) or between those of the patients with and without sarcopenia (-12.58 ± 5.04 vs. -14.65 ± 2.98; *p* = 0.74).

**Table 5:** Global health covariation during the first chemotherapy cycle in patients with and without sarcopenia and in patients with and without cachexia (N = 60).

Group	n (%)	Global health covariation		Adjusted delta Mean ± SD	P
		Pre-chemo Mean ± SD	Post-chemo Mean ± SD		
With cachexia	33 (55.0)	71.97 ± 15.28	56.56 ± 25.49	-15.39 ± 3.52	0.61
Without cachexia	27 (45.0)	71.30 ± 15.56	58.64 ± 25.79	-12.68 ± 3.90	
With sarcopenia	14 (23.3)	73.21 ± 20.46	60.71 ± 27.82	-12.58 ± 5.04	0.74
Without sarcopenia	46 (76.7)	71.20 ± 13.57	56.52 ± 24.90	-14.65 ± 2.98	

Pre-chemo, before the first cycle of chemotherapy; Post-chemo, after the first cycle of chemotherapy.

## DISCUSSION

Our results show that there was a reduction in QoL after the first cycle of chemotherapy, but changes with no difference between patients with changes in their muscle mass. In agreement with a previous report^(21)^, we found that 45 (75.0%) of our patients experienced some level of self-reported toxicity during their initial chemotherapy cycle, which was directly linked to cachexia and sarcopenia. These findings are critical, because they highlight the urgent need for integrating muscle mass evaluation into the standard clinical assessment of cancer patients. Such measures can significantly influence treatment decisions, highlighting the importance of personalized therapeutic strategies based on a detailed body composition analysis^(22)^.

There is growing evidence of an association between sarcopenia and cachexia, which tend to worsen overall survival rates in patients suffering from gastrointestinal cancer^(23)^. As in other studies, we observed a reduction in QoL in some of the EORTC QLQ-C30 domains, regardless of the presence or absence of sarcopenia and cachexia. Our findings are consistent with the systematic review conducted by Zhao et al.^(24)^ which evaluated patients with breast cancer and showed that patient QoL declined during chemotherapy. These results are important when advising patients about side effects of the disease and the necessity of paying greater attention to the symptoms of cancer related to cachexia and sarcopenia, such as fatigue, weakness of limbs, and loss of hair.

Our study adds relevant information to the growing body of evidence of associations between muscle mass and QoL in individuals with cancer. However, there is still a lack of prospective longitudinal studies evaluating the interaction between muscle mass and QoL over time.

Ryan et al.^(25)^ emphasized the significance of diminished muscle mass and reduced muscle attenuation, which correlate with a lower tolerance to chemotherapy, a substantial decline in patient performance status and QoL, and reduced survival prospects. Fearon et al.^(26)^ evaluated 170 patients with advanced pancreatic cancer, among whom 102 (60%) had cachexia, and found that QoL scores were significantly lower among the patients with cachexia than among those without. In a separate study, involving 135 patients with non-small cell lung cancer, Stene et al.^(27)^ found no significant difference in QoL between the patients with sarcopenia and those without. These variations in study outcomes underscore the need for assessing QoL among cancer patients across various cancer types and considering their diverse health statuses and body compositions.

Our study provides new data about the relationships that cachexia and sarcopenia have with QoL in patients with gastrointestinal cancer who have recently started chemotherapy, demonstrating a significant reduction in almost all “general” domains of QoL, resulting in an overall loss of QoL during chemotherapy. Physical function was one of the most affected domains during the treatment, which aligns with prior research demonstrating that sarcopenia and cachexia are linked to lower tolerance to chemotherapy, substantial declines in performance status, reduced QoL, and poor survival outcomes^(25)^. This decrease in QoL domains can also be explained by the high toxicity experienced by the studied patients, reported previously and directly associated with cachexia and sarcopenia. These conditions directly impact muscle force^(19)^, which is critical for maintaining functional capacity. Maintaining patients above critical thresholds of muscle mass might be correlated with substantial clinical advantages^(28)^.

By aligning the objectives with a focus on the importance of CT, our intention was to provide a solid foundation for understanding and addressing the challenges faced by cancer patients, establishing a connection between early detection of these conditions and interventions aimed at improving QoL during chemotherapy.

Sarcopenia has been associated with mortality and it has been reported to be a significant predictor of toxicity increase of treatment and time reduction of tumor progression in patients with cancer. The presence of reduced muscle mass appears to be a marker of increased morbidity and mortality, diminished physical function, and lower QoL. Therefore, maintaining patients above these critical thresholds might be correlated with substantial clinical advantages^(28)^.

In our analysis of scores at different time points, no significant variation was observed in the social functioning domain of the EORTC QLQ-C30. That domain encompasses factors related to anxiety and the interactions of the patient with family and friends. Several factors, such as age, could have contributed to a decline in the social aspects of QoL. The average age of the subjects in the present study was 60.9 years. Older adults are more prone to emotional decline and decline in their social relationships due to a loss of autonomy. Dilution of physical functioning may suggest this, in addition to reinforcing a sense of overload for the family, in addition to the disease itself contributing to such feelings. During treatment, aspects like well-being and QoL may be adversely affected by cachexia, with patients potentially suffering from fatigue, weakness, loss of appetite, increased inflammatory markers, decreased tolerance to treatment, and a generally poorer prognosis^(29)^. Therefore, it is becoming critically important to assess the QoL of patients undergoing chemotherapy protocols. By aligning the objectives with a focus on the importance of CT, our intention was to provide a solid foundation for understanding and addressing the challenges faced by cancer patients, establishing a connection between early detection of these conditions and interventions aimed at improving QoL during chemotherapy.

Our study has some limitations. The diversity among primary cancer sites, cancer stages, and their corresponding chemotherapy protocols constitutes a notable limitation. In addition, the lack of subgroup analysis could influence the interpretation of the results. Our study was also constrained by the lack of repeated evaluations following chemotherapy cycles. It is recognized that patients experience a decline in QoL during chemotherapy due to the toxic effects of chemotherapy drugs. However, these effects may diminish post-chemotherapy, potentially leading to an improvement in QoL, an aspect not captured in our analysis.

In conclusion, the present study highlighted the importance of assessing sarcopenia and cachexia in patients undergoing chemotherapy. Although our results did not show a significant difference between patients with sarcopenia or cachexia and those with neither, in terms of the decline in QoL, the evaluation of muscle mass with CT could still play a crucial role in guiding personalized treatment strategies, because sarcopenia and cachexia have been associated with decreased tolerance to treatment and poorer prognosis. Therefore, integrating the evaluation of muscle mass into radiology reports could provide valuable insights for intervention planning in cancer care. Finally, we highlight the importance of using CT to evaluate body composition in the monitoring of cancer patients. The systematic inclusion of these results in radiology reports emerges as a crucial step. Collecting information about musculoskeletal health could not only make for more comprehensive diagnostics but could also play a fundamental role in guiding personalized intervention strategies. Therefore, we encourage our fellow radiologists to consider incorporating these data into their reports, recognizing its direct impact on QoL and on the planning of specific care for patients in cancer treatment.
